# Estrogen pathway polymorphisms in relation to primary open angle glaucoma: An analysis accounting for gender from the United States

**Published:** 2013-07-12

**Authors:** Louis R. Pasquale, Stephanie J. Loomis, Robert N. Weinreb, Jae H. Kang, Brian L. Yaspan, Jessica Cooke Bailey, Douglas Gaasterland, Terry Gaasterland, Richard K. Lee, William K. Scott, Paul R. Lichter, Donald L. Budenz, Yutao Liu, Tony Realini, David S. Friedman, Catherine A. McCarty, Sayoko E. Moroi, Lana Olson, Joel S. Schuman, Kuldev Singh, Douglas Vollrath, Gadi Wollstein, Donald J. Zack, Murray Brilliant, Arthur J. Sit, William G. Christen, John Fingert, Peter Kraft, Kang Zhang, R. Rand Allingham, Margaret A. Pericak-Vance, Julia E. Richards, Michael A. Hauser, Jonathan L. Haines, Janey L. Wiggs

**Affiliations:** 1 Department of Ophthalmology, Mass Eye & Ear Infirmary, Harvard Medical School, Boston, MA; 2 Channing Division of Network Medicine, Harvard Medical School, Boston, MA; 3 Department of Ophthalmology and Hamilton Glaucoma Center, University of California, San Diego, SD, CA; 4 Genentech Inc, San Francisco, CA; 5 Center for Human Genetics Research, Vanderbilt University School of Medicine, Nashville TN; 6 Eye Doctors of Washington, Chevy Chase, MD; 7 Scripps Genome Center, University of California at San Diego, San Diego, CA;; 8 Bascom Palmer Eye Institute and Hussman Institute for Human Genomics, University of Miami Miller School of Medicine, Miami, FL; 9 Department of Ophthalmology and Visual Sciences, University of Michigan, Ann Arbor, MI;; 10 Department of Ophthalmology, University of North Carolina, Chapel Hill, NC; 11 Department of Ophthalmology, Duke University Medical Center, Durham, NC; 12 Medicine, Duke University Medical Center, Durham, NC; 13 Department of Ophthalmology, West Virginia University Eye Institute, Morgantown, WV;; 14 Wilmer Eye Institute, Johns Hopkins University Hospital, Baltimore, MD; 15 Essentia Institute of Rural Health, Duluth, MN; 16 Department of Ophthalmology, UPMC Eye Center, University of Pittsburgh, Pittsburgh, PA;; 17 Department of Ophthalmology, Stanford University, Palo Alto, CA; 18 Department of Genetics, Stanford University, Palo Alto, CA; 19 Center for Human Genetics, Marshfield Clinic Research Foundation, Marshfield, WI; 20 Department of Ophthalmology, Mayo Clinic, Rochester, MN; 21 Department of Medicine, Brigham and Women’s Hospital, Boston, MA; 22 Departments of Ophthalmology and Anatomy/Cell Biology, University of Iowa, College of Medicine, Iowa City, IO;; 23 Department of Biostatistics, Harvard School of Public Health, Boston, MA.

## Abstract

**Purpose:**

Circulating estrogen levels are relevant in glaucoma phenotypic traits. We assessed the association between an estrogen metabolism single nucleotide polymorphism (SNP) panel in relation to primary open angle glaucoma (POAG), accounting for gender.

**Methods:**

We included 3,108 POAG cases and 3,430 controls of both genders from the Glaucoma Genes and Environment (GLAUGEN) study and the National Eye Institute Glaucoma Human Genetics Collaboration (NEIGHBOR) consortium genotyped on the Illumina 660W-Quad platform. We assessed the relation between the SNP panels representative of estrogen metabolism and POAG using pathway- and gene-based approaches with the Pathway Analysis by Randomization Incorporating Structure (PARIS) software. PARIS executes a permutation algorithm to assess statistical significance relative to the pathways and genes of comparable genetic architecture. These analyses were performed using the meta-analyzed results from the GLAUGEN and NEIGHBOR data sets. We evaluated POAG overall as well as two subtypes of POAG defined as intraocular pressure (IOP) ≥22 mmHg (high-pressure glaucoma [HPG]) or IOP <22 mmHg (normal pressure glaucoma [NPG]) at diagnosis. We conducted these analyses for each gender separately and then jointly in men and women.

**Results:**

Among women, the estrogen SNP pathway was associated with POAG overall (permuted p=0.006) and HPG (permuted p<0.001) but not NPG (permuted p=0.09). Interestingly, there was no relation between the estrogen SNP pathway and POAG when men were considered alone (permuted p>0.99). Among women, gene-based analyses revealed that the catechol-O-methyltransferase gene showed strong associations with HTG (permuted gene p≤0.001) and NPG (permuted gene p=0.01).

**Conclusions:**

The estrogen SNP pathway was associated with POAG among women.

## Introduction

There is accumulating evidence that circulating estrogen levels influence processes relevant to glaucoma in the reproductive and post-reproductive years [1]. For example, elevated estrogen levels in pregnancy were associated with an approximate 10% reduction in intraocular pressure (IOP) [2-4], despite a concomitant increase in central corneal thickness [5]. Estrogen levels throughout the normal menstrual cycle influence the neuroretinal rim area measured with confocal scanning laser ophthalmoscopy [6] and mean sensitivity on short wavelength automated perimetry [7] in healthy women without ocular disease. Postmenopausal women have higher IOP than age-matched women who are still menstruating [2], and postmenopausal hormone (PMH) use is associated with reduced IOP in several studies [8-13]. Furthermore, ocular blood flow is reduced with aging, and PMH use seems to restore ocular blood flow resistance indices to levels achieved during the premenopausal period [9,14]. Early age at menopause was associated with increased risk of open angle glaucoma in the Rotterdam study [15]. In the Nurses Health Study (NHS), among women aged 65 or older, entering menopause at age 54 or later was associated with reduced risk of primary open angle glaucoma (POAG) [16]. Furthermore, the NHS revealed that PMH use was also associated with a reduced risk of high-tension POAG among women 65 years of age or older [16]. Collectively, these data suggest that estrogen levels in women may influence POAG, an age- and IOP-related optic neuropathy. Conversely, little is known about the role, if any, sex hormones play in POAG pathogenesis among men.

To date, genetic studies of estrogen’s role in POAG have been restricted to candidate gene studies. For example, given that estrogen receptor 2 (*ESR2*) is present in retinal ganglion cells of men and women [17], investigators have studied whether estrogen receptor 1 (*ESR1*) and *ESR2* single nucleotide polymorphisms (SNPs) are associated with POAG with conflicting results [18,19]. No group has assessed whether an entire set of estrogen metabolic pathway SNPs is associated with POAG using a gender-specific approach. We hypothesize that if estrogen levels influence glaucoma risk, then a relationship may exist between the aggregate of estrogen pathway SNPs and POAG. This strategy allows for detection of a signal that arises from multiple individual weak genetic effects that impact the glaucomatous process. Researchers have studied the aggregate effects of genetic variants in the estrogen metabolic pathway for association with breast [20] and uterine cancer [21], and these approaches have yielded encouraging results. We assembled high throughput genotyping SNP data from two large data sets—the Glaucoma Genes and Environment (GLAUGEN) study and the National Eye Institute Glaucoma Human Genetics Collaboration (NEIGHBOR) consortium—into an estrogen metabolic SNP panel and used the Pathway Analysis by Randomization Incorporating Structure (PARIS) analysis software package [22] to test if this panel is associated with POAG. Similarly, we assessed whether collections of SNPs within genes that comprise the pathway are associated with POAG. Since the association between *ESR2* SNPs and POAG [18,19] and other complex diseases [23,24] may differ between men and women, we conducted our analyses with consideration of such possible gender differences.

## Methods

### Description of the study populations

The GLAUGEN study is part of a network of collaborative studies [25] that has contributed to an improved understanding of genotype-phenotype associations [26] and gene–environment interactions for various complex traits [27], including POAG [28,29]. The GLAUGEN study contains POAG cases and controls derived from the Genetic Etiologies of Primary Open-Angle Glaucoma (GEP) study, the Nurses’ Health Study (NHS), and the Health Professionals Follow-up Study (HPFS). The GEP is a clinical case-control study from Massachusetts Eye and Ear Infirmary while the NHS and HPFS contain cases and controls nested within population-based cohorts at risk for POAG. Details regarding the inclusion and exclusion criteria for the GLAUGEN POAG case-control group have been described at the database of Genotypes and Phenotypes (dbGaP), accession number phs000308.v1.p1, and by Wiggs et al. [30]. The institutional review boards of Massachusetts Eye and Ear Infirmary, Brigham and Women’s Hospital, and Harvard School of Public Health approved this study.

The NEIGHBOR consortium represents the collaborative efforts of 12 institutions where POAG cases and controls were collected using harmonized criteria. Information pertaining to the study sites, design features, inclusion criteria, and clinical variables collected in the NEIGHBOR consortium has been described [31]. Data from the NEIGHBOR consortium have contributed to the understanding of the genetic structure of POAG [29] and central corneal thickness [32,33]. The institutional review boards of the participating institutions also approved this study.

### Ophthalmic characteristics of study participants

For the patients, slit-lamp biomicroscopy did not reveal exfoliation syndrome, pigment dispersion syndrome, or other findings that could produce elevated IOP. The iridocorneal filtration angle was judged to be open in both eyes. At least one eye had a cup-disc ratio (CDR) >0.7 and visual field (VF) loss or the VF loss was replicated on a subsequent test regardless of CDR. Eyes with VF loss had defects localized to the nerve fiber layer on reliable tests (fixation loss was ≤33%, false positive rate was ≤20%, and false negative rate was ≤20%). The type of VF test used was not specified. IOP at diagnosis was collected and used to categorize the POAG cases into high-pressure glaucoma (HPG with IOP ≥22 mmHg) and normal pressure glaucoma (NPG with IOP <22 mmHg) subtypes. Controls underwent an eye exam that revealed an IOP <22 mmHg and CDR≤0.6 in both eyes. For members of the NHS and HPFS, the eye exam was performed by the participants’ local eye care provider, and that exam reportedly did not reveal signs of glaucoma.

### Genotyping data

The methods used to collect, extract, and plate DNA for GLAUGEN [30] and NEIGHBOR [29] have been previously outlined. Genotyping for GLAUGEN and NEIGHBOR was performed at the Broad Institute (Cambridge, MA) and the Center for Inherited Disease Research (Baltimore, MD), respectively. We used the Illumina Human660W-Quad-v1 array (Illumina; San Diego, CA) to genotype subjects for both studies. The algorithm for calling genotypes, the SNP quality control (QC) filters used, and the preliminary analyses undertaken to discover the predictors of genotype calling rates have been discussed in prior publications [29,30].

### Gene association analyses

We used PLINK v1.07 [34] to perform gene association analysis for POAG in GLAUGEN and NEIGHBOR for SNPs that passed the QC process (484,419 SNPs in GLAUGEN and 521,683 SNPs in NEIGHBOR) as previously described [29,30]. Briefly, these analyses were conducted among self-reported Caucasians and adjusted for age, gender, study site, and population structure in both study groups. In GLAUGEN, additional adjustments were made for the DNA extraction method (DNAzol, Beverly, MA; Qiagen or GENTRA, Germatown, MD) and the DNA source (blood or cheek cell sample) because these parameters influenced the genotyping call rates [29,30]. Using the METAL software package [35], we then performed a meta-analysis of the GLAUGEN and NEIGHBOR data sets and obtained p values and odds ratios for the 480,335 overlapping SNPs in the combined data set that passed QC. In this paper, we used the p values and odds ratio for the estrogen metabolism SNPs in the pathway analysis discussed below.

### Defining the estrogen pathway and Pathway Analysis by Randomization Incorporating Structure pathway analysis

**Figure 1:**
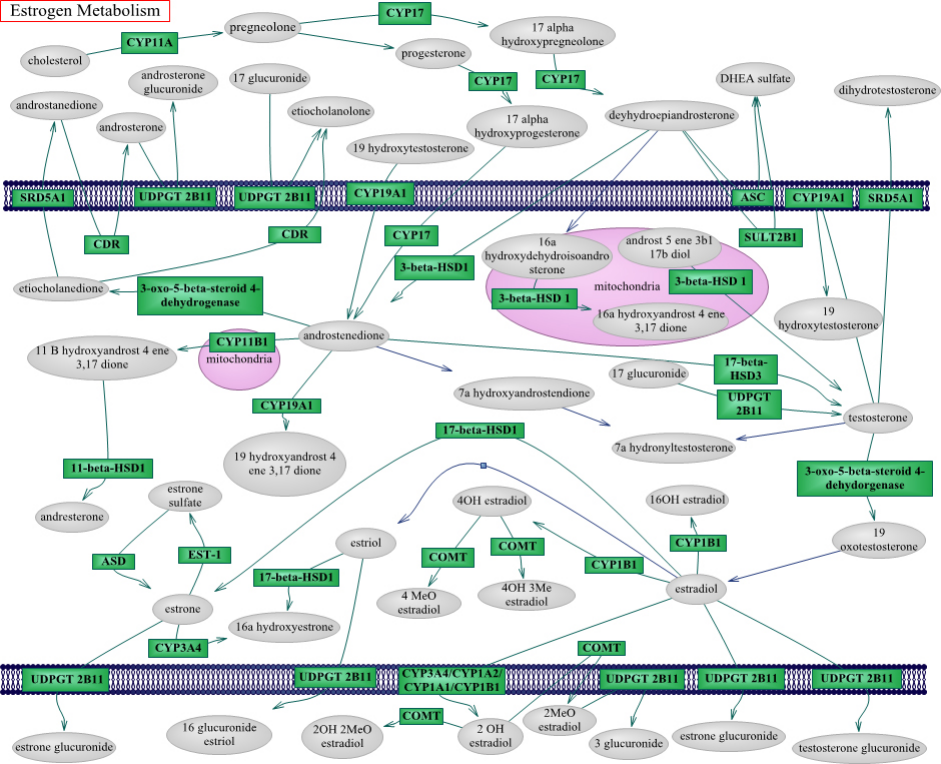
The estrogen metabolic pathway is depicted. The top and bottom sections represent the extracellular space, and the middle section represents the intracellular space. The mitochondrial compartment is labeled in pink. Enzymes are labeled in green and estrogen metabolites are labeled in grey. Protein definitions with corresponding abbreviations and their associated genes are as follows: CDR=Aldo-keto reductase family 1 member C4 (Chlordecone reductase), coded by *AKR1C4*; 3-oxo-5-beta-steroid 4-dehydrogenase=3-oxo-5-beta-steroid 4-dehydrogenase, coded by *AKR1D1*; ASD=Arylsulfatase D, coded by *ARSD*; COMT=catechol-O-methyltransferase, coded by *COMT*; CYP11A=cytochrome P450, family 11, subfamily A, polypeptide 1, coded by *CYP11A1*; CYP11B1=cytochrome P450, family 11, subfamily B, polypeptide 1, coded by *CYP11B1*; CYP17=cytochrome P450, family 17, subfamily A, polypeptide 1, coded by *CYP17A1*; CYP19A1=Cytochrome P450 19A1, coded by *CYP19A1*; CYP1A1=cytochrome P450, family 1, subfamily A, polypeptide 1, coded by *CYP1A1*; CYP1A2=cytochrome P450, family 1, subfamily A, polypeptide 2, coded by *CYP1A2*; CYP1B1=Cytochrome P450 1B1, coded by *CYP1B1*; CYP3A4=cytochrome P450, family 3, subfamily A, polypeptide 4, coded by *CYP3A4*; 11-beta-HSD1=Corticosteroid 11-beta-dehydrogenase isozyme 1 (11-beta-hydroxysteroid dehydrogenase 1), coded by *HSD11B1*; 17-beta-HSD1=Estradiol 17-beta-dehydrogenase 1 (17-beta-hydroxysteroid dehydrogenase type 1), coded by *HSD17B1*; 17-beta-HSD3=hydroxysteroid (17-beta) dehydrogenase 3, coded by *HSD17B3*; 3-beta-HSD1=3 beta-hydroxysteroid dehydrogenase/Delta 5-->4-isomerase type 1, coded by *HSD3B1*; SRD5A1=steroid-5-alpha-reductase, alpha polypeptide 1 (3-oxo-5 alpha-steroid delta 4-dehydrogenase alpha 1), coded by *SRD5A1*; ASC=Steryl-sulfatase (Arylsulfatase C), coded by *STS*; EST-1=Estrogen sulfotransferase, coded by *SULT1E1*; SULT2B1=Sulfotransferase family cytosolic 2B member 1, coded by *SULT2B1*; UDPGT 2B11=UDP-glucuronosyltransferase 2B11, coded by *UGT2B11.* Two genes not in the figure but in the pathway analysis are ESR1=estrogen receptor 1, coded by *ESR1*; ESR2=estrogen receptor 2 (ER beta), coded by *ESR2*. Single nucleotide polymorphisms for these genes are derived from the Illumina platform array. Additional abbreviations for estrogen metabolites are as follows: OH=hydroxyl; Me=methoxy

We generated a custom list of 903 SNPs in 23 genes across 15 chromosomes comprising the estrogen metabolic pathway using the Kyoto Encyclopedia of Genes and Genomes (KEGG) [36] online database and other academic sources [37-40] (see Figure 1). In analyses in which men and women were considered together or men were considered alone, X chromosome variants involved in estrogen metabolism were excluded, yielding a total of 874 SNPs available for study. We uploaded the meta-analyzed p values for genotyped SNPs within these genes ± a 50kB genomic window to PARIS. The statistical approach used by PARIS has been previously described [22]. Conceptually, PARIS first analyzes the genomic features of the pathway, namely, the number of linkage disequilibrium (LD) blocks, the number of SNPs per block, and the number of SNPs not in any LD block for the gene biomarker assembly. The estrogen pathway contains 122 complex features (number of LD blocks with two or more types SNPs) and 123 simple features (number of SNPs not in any LD block). After this step, PARIS compares the overall association between the estrogen pathway and POAG versus the associations between 1,000 randomly generated pathways with similar genetic architecture and POAG. PARIS assesses pathways of interest using a permuted p value threshold of <0.05, which is considered statistically significant. For example, with the estrogen pathway among women, the results indicated 39 significant features; specifically, 15 of 123 simple features (or SNPs) had p value<0.05 for association with POAG, and 24 of 122 complex features were statistically significant in relation to POAG (a complex feature was significant if any SNP in the LD block had a p value<0.05). The PARIS-generated permuted p value=0.006 for the estrogen pathway among women indicated that six of 1,000 random pathways with similar genetic architecture had a higher significant feature count (>39 significant features with p<0.05) in relation to POAG. These analyses were performed for POAG in a gender-specific manner. Subsequently, we considered gender-specific HPG and NPG as outcomes. Finally, we looked at these outcomes when men and women were considered together. To determine which of the genes or SNPs in the estrogen metabolism pathway contributed to any significant signal in the pathway overall, we used the “-I” (Investigate) option within PARIS, which reports the p values of genes and SNPs within the pathway.

## Results

This study sample included 3,108 cases (976 from GLAUGEN and 2,132 from NEIGHBOR) and 3,430 controls (1,140 from GLAUGEN and 2,290 from NEIGHBOR; Table 1). The estrogen SNP pathway was associated with POAG overall (permuted p=0.006) and HPG (permuted p<0.001) but not NPG (permuted p=0.09) among women. Among men, the relation between the estrogen SNP pathway and POAG (permuted p>0.99), HPG (permuted p=0.92), and NPG (permuted p=0.59) was consistently null across the IOP spectrum. The estrogen pathway was not significantly associated with POAG overall (permuted p=0.72), HPG (permuted p=0.12), and NPG (permuted p=0.06) when men and women were considered together.

**Table 1:** Significance of the estrogen pathway in relation to primary open-angle glaucoma (POAG) in the combined GLAUGEN and NEIGHBOR dataset for overall POAG, and subtypes of POAG defined by intraocular pressure at diagnosis, overall and by gender.

	N		Mean age (years)		Estrogen pathway p-value
	Cases	Controls	Cases	Controls	
Overall	3108	3430	65.7	67.7	0.72
Males only	1426	1493	65.6	68.4	>0.99
Females only	1682	1937	65.7	67.3	**0.006**
HPG	1637	3430	61.8	67.7	0.12
NPG	717	3430	65.5	67.7	0.06
HPG, females only	859	1937	61.8	67.3	**<0.001**
NPG, females only	419	1937	65.1	67.3	0.09
HPG, males only	778	1493	61.8	68.4	0.92
NPG, males only	298	1493	66.1	68.4	0.59

P-values are permuted p-values. P-values <0.05 are in highlighted in bold. Abbreviations: GLAUGEN=the Glaucoma Genes and Environment study; NEIGHBOR =National Eye Institute Glaucoma Human Genetics Collaboration; HPG=High pressure glaucoma; NPG=Normal pressure glaucoma. NB: The number of HPG and NPG do not add up to 3108 because for some POAG patients the IOP at diagnosis was not known.

Analogous to the pathway approach, we assessed the aggregate of SNPs within specific estrogen pathway genes for association with POAG. We performed this analysis to gain insight into which aspects of the pathway contributed to the association we observed among women. *ESR1*, aldo-keto reductase family 1, member D1 (*AKR1D1*), cytochrome P450, family 3, subfamily A, polypeptide 4 (*CYP3A4*), catechol-O-methyltransferase (*COMT*), and steroid sulfatase (microsomal), isozyme S (*STS*) were significantly (permuted gene p values of <0.001, 0.05, 0.01, <0.001, and <0.001, respectively) associated with POAG overall among women (Table 2). With the exception of *AKR1D1*, these genes were also associated with HPG among women (all with permuted gene p<0.001), along with aldo-keto reductase family 1, member C4 (*AKR1C4*; permuted gene p=0.002) and hydroxysteroid (17-beta) dehydrogenase 1 (*HSD17B1*; permuted p<0.001). Only *COMT* and *STS* were associated with HPG (permuted p<0.001 for both genes) and NPG (permuted gene p=0.01 and p<0.001, respectively); otherwise, there was little overlap between the genes significantly associated with HPG and NPG. Other genes associated with NPG among women included *AKR1D1* (permuted gene p=0.046), *CYP17A1* (permuted gene p=0.04), *ESR2* (permuted gene p<0.001), *CYP1A1* (permuted gene p<0.001), and *CYP1A2* (permuted gene p=0.004).

**Table 2:** Gene significance within the estrogen pathway among women, subdivided by intraocular pressure at the time of glaucoma diagnosis.

**Gene**	**Chr**	**# of simple features**	**# of complex features**	**Gene p-value^1^, POAG overall**	**Gene p-value^1^,** **HPG**	**Gene p-value^1^,** **NPG**
HSD11B1	1	6	7	>0.99	>0.99	0.31
HSD3B1	1	2	3	>0.99	>0.99	0.07
CYP1B1	2	1	7	0.29	>0.99	>0.99
SULT1E1	4	2	2	>0.99	>0.99	0.07
UGT2B11	4	2	1	>0.99	>0.99	>0.99
SRD5A1	5	12	6	0.06	0.49	0.45
ESR1	6	22	20	**< 0.001**	**< 0.001**	0.39
AKR1D1	7	10	7	**0.05**	>0.99	**0.046**
CYP3A4	7	4	2	**0.01**	**< 0.001**	>0.99
CYP11B1	8	1	2	>0.99	0.07	>0.99
HSD17B3	9	4	8	>0.99	>0.99	0.50
AKR1C4	10	2	7	>0.99	**0.002**	>0.99
CYP17A1	10	0	5	>0.99	>0.99	**0.04**
ESR2	14	2	7	0.11	0.10	**< 0.001**
CYP11A1	15	10	4	0.35	>0.99	0.34
CYP19A1	15	15	11	0.44	0.09	0.72
CYP1A1	15	1	2	>0.99	>0.99	**< 0.001**
CYP1A2	15	2	3	>0.99	0.21	**0.004**
HSD17B1	17	0	2	0.06	**< 0.001**	>0.99
SULT2B1	19	10	4	>0.99	0.07	>0.99
COMT	22	11	11	**< 0.001**	**< 0.001**	**0.01**
ARSD	X	4	0	>0.99	>0.99	>0.99
STS	X	0	1	**< 0.001**	**< 0.001**	**< 0.001**

See Figure 1 for the gene names. All p-values are permuted p-values as discussed in the methods. P values <0.05 are in bold. Simple features refer to SNPs not in any LD block. Complex features refers to LD blocks with 2 or more types SNPs. Abbreviations: Chr=chromosome; HPG=high pressure glaucoma; NPG=normal pressure glaucoma.

Although the *COMT* SNP set showed a significant association with HPG and NPG among women, the SNP set was not associated with the POAG or POAG subtypes among men (permuted gene p≥0.66). Among men, gene-based analysis revealed one gene associated with POAG overall and with HPG: *CYP1B1* (permuted gene p≤0.02). The only other gene associated with HPG among men was *CYP11A1* (permuted gene p=0.02). Furthermore, the following estrogen metabolism genes were associated with NPG among men: *HSD11B1* (permuted gene p=0.003), *CYP1A1* (permuted gene p=0.001), and *CYP1A2* (permuted gene p<0.001).

To refine the source of significant association between estrogen metabolic pathways and POAG even further among women, we report the nominally significant SNPs that were in any relevant genes in POAG overall, HPG or NPG (Appendix 1). The p values for all SNPs in the estrogen metabolic pathway are provided in Appendix 2. As with the genes overall, there was some overlap between the significant SNPs in POAG overall and HPG, but little overlap between the HPG and NPG subsets. For example, in *COMT*, one of two genes that were significant in HPG and NPG, only one significant SNP was shared between the HPG and NPG subsets (rs3804047). Although one *COMT* SNP (rs2531697) showed particularly strong p values for association with HPG in women (p=4.10E-05), the SNP would not remain significant if one accounted for the multiple comparisons made in this analysis (903 SNPs stratified by gender and IOP yielding a corrected significance level of 1.85E-05); however, our pathway analysis points to a large number of *COMT* SNPs significant for POAG and its subtypes among women.

## Discussion

The relationship between environmental factors such as PMH use and POAG among women has been previously described [15,16,41]. Candidate gene studies assessing selected estrogen metabolic SNPs [19,42] as well as candidate gene–environment interactions [43,44] in POAG among women have also been reported. This is the first paper to assess a possible association between the estrogen metabolic SNP pathway as a whole and POAG using genome-wide association data. Our results suggest that the estrogen metabolic pathway is associated with POAG among women supporting the notion that multiple genetic signals of modest effect contribute to the glaucomatous process. Furthermore, the association between the estrogen metabolic pathway and POAG among women was more significant in those classified as having HPG. The results highlight a distinct sexual dimorphism in that the pathway is definitively not associated with POAG among men. This latter result also contributes to a null result when the relation between the estrogen pathway and POAG is considered in men and women jointly.

*ESR1* codes for estrogen receptor alpha and is expressed in the retina of both sexes [17]. In a gene association study of 87 incident POAG cases and 3,616 controls, a haplotype of two *ESR1* SNPs was not associated with open angle glaucoma in women or men in the Rotterdam study [19]. Our study with 3,108 cases and 3,430 controls contains genotypes on 127 *ESR1* SNPs. Collectively, *ESR1* SNPs contributed to the association between the estrogen pathways and POAG overall and in HPG in women (see Table 2; gene permuted p<0.001). The signal from *ESR1* was modest as the *ESR1* gene permuted p value for association with POAG among women was 0.31 in NEIGHBOR alone (2132 cases) and 0.26 in GLAUGEN alone (976 cases), only reaching significance in the combined data set. Furthermore, *ESR1* was modestly associated with POAG overall when men and women were considered together (gene permuted p=0.023).

Exogenous estrogen is protective in various animal models of neurodegeneration [45,46] including experimental models of glaucoma [47,48]. This raises questions regarding whether polymorphisms in *ESR2* (which codes for the estrogen receptor found on retinal ganglion cells [17]) may alter affinity for estrogen and affect retinal ganglion cell viability in glaucoma. A Japanese group found an association between *ESR2* rs1256031 and rs4986938 variants and HPG in a study including 212 female cases and 191 controls [42]. Our study included 859 women with HPG and 1,937 female controls and contained genotypes on 38 *ESR2* SNPs including several SNPs in high LD with rs1256031 and rs4986938. Collectively, we found that *ESR2* SNPs were associated with NPG (gene permuted p<0.001) but not HPG (gene permuted p=0.10) among women. Furthermore, the rs1256031 and rs4986938 variants were specifically not associated with HPG among the women in our data set. The Rotterdam study of European Caucasians did not find associations between these two *ESR2* SNPs and open angle glaucoma in women but found an association in men. We did not find an association between the *ESR2* collection of SNPs and POAG in men (gene permuted p>0.99) or for the rs1256031 and rs4986938 SNPs in particular (p≥0.45). The conflicting results may be due to differences in sample size and population stratification. Assessing large data sets and evaluating the collective effects of genes in a pathway and the pathway as a whole in relation to glaucoma provide greater power to find existing associations. Overall, our data set suggests that estrogen receptor polymorphisms are important in POAG among women, with *ESR1* SNPs playing an important role in HPG and *ESR2* SNPS playing a role in NPG.

*COMT* codes for catechol-O-methyltransferase that catalyzes the transfer of a methyl group from S-adenosyl methionine to a hydroxyl group on estrogen, dopamine, and epinephrine. The enzyme COMT is involved in methylating two derivatives of estradiol—2-hydroxyestradiol and 4-hydroxyestradiol—and perhaps reducing estrogen bioavailability. Variants in *COMT* were associated with HPG and NPG in women but not men. Although no other group has reported on associations between *COMT* variants and open angle glaucoma, we saw some evidence of internal replication within our data sets. Among women, the permuted p value for association between *COMT* and HPG was 0.01 in NEIGHBOR and 0.02 in GLAUGEN. Interestingly, there is robust evidence that gene variants in *COMT* differentially impact brain function in men and women [20,49-55].

Cytochrome P450 enzymes have an important role in estrogen metabolism, and several genes coding for these liver enzymes contributed to the association between the estrogen pathway and POAG in women. *CYP3A4* showed an association with HTG in women while *CYP17A1*, *CYPIA1*, and *CYPIA2* showed an association with NPG among women. Selected *CYP1B1* SNPs have been studied in association with POAG. A prior meta-analysis of the *CYP1BI* SNPs in association with POAG consisting of 1,953 cases and 1,341 controls that found no significant association [56] is largely consistent with our larger study (3,108 cases and 3,430 controls), which found no association between the *CYP1B1* aggregate of SNPs and POAG among men and women together (permuted gene p=0.098). The role of cytochrome P450 metabolism in glaucoma is unknown, but it is intriguing to speculate that metabolism of intracellular estrogen is involved. Members of the cytochrome P450 family could alter estrogen metabolism over time [57] and impact the development of open angle glaucoma.

This study has limitations. First, none of the individual SNPs in the estrogen metabolic pathway were significantly associated with POAG either overall or in either gender if a strict Bonferroni correction is imposed. Nonetheless, existing genome-wide studies of POAG are underpowered to find biologically meaningful SNPs of modest effects due to the need to curb the false discovery rate. The purpose of this analysis is to use a hypothesis-driven approach that involves SNP subsets to find potentially important associations that provide insight into POAG pathogenesis. Second, no other current data set of comparable size can be used to replicate the findings we present here; however, we demonstrated internal replication between NEIGHBOR and GLAUGEN for our one novel finding, the association between *COMT* and HPG in women. Therefore, even though it is intriguing that common polymorphisms in a network of estrogen-metabolizing genes may be relevant to POAG development among women, caution is warranted until these findings can be replicated in other large data sets. Finally, although the estrogen pathway SNPs were associated with POAG overall in women, it is not possible to identify functional networks with discrete effect size on the glaucomatous process. Such analysis is the focus of future research.

This is the largest study (6,538 subjects) assessing the relation between aggregate effects of 903 estrogen-metabolizing SNPs in relation to POAG. We find that this SNP collection is associated with POAG in women but not men. These data may shed light on the many epidemiological data and basic science research implicating estrogen levels in POAG pathogenesis among women. The work may open new avenues of research into the role of estrogen in the development of POAG and may lead to new gender-specific prevention strategies that are tailored to an individual’s genetic makeup.

## Appendix 1.

Significant SNP p values with odds ratio (OR) from the combined GLAUGEN-NEIGHBOR dataset for genes in the estrogen metabolic pathway that were significantly associated with POAG overall or with its subtypes among women. To access the data, click or select the words “Appendix 1.”

## Appendix 2.

Single nucleotide polymorphism (SNP) p values with odds ratios (OR) from the combined GLAUGEN-NEIGHBOR dataset for genes in the estrogen metabolic pathway among women. To access the data, click or select the words “Appendix 2.”
